# Sex-Specific and State-Dependent Neuromodulation Regulates Male and Female Locomotion and Sexual Behaviors

**DOI:** 10.34133/research.0321

**Published:** 2024-02-22

**Authors:** Xinyu Jiang, Mengshi Sun, Jie Chen, Yufeng Pan

**Affiliations:** ^1^ The Key Laboratory of Developmental Genes and Human Disease, School of Life Science and Technology, Southeast University, Nanjing 210096, China.; ^2^ Co-innovation Center of Neuroregeneration, Nantong University, Nantong 226019, China.

## Abstract

Males and females display dimorphic behaviors that often involve sex-specific locomotor patterns. How the sexually dimorphic locomotion is mediated is poorly understood. In this study, we identify a neuropeptide that oppositely regulates locomotion for efficient sexual behaviors in *Drosophila* males and females. We find that males are less active than females if isolated. However, when sexually aroused through activating homologous but sexually dimorphic pC1 neurons, males exhibit higher activity levels than females. We discover diuretic hormone 44 (DH44) that functions in pC1 neurons in a sex-specific way to inhibit male locomotion and promote female locomotion. Surprisingly, DH44 exerts opposite effects in sexually aroused flies to promote male locomotion and suppress female locomotion, which is crucial for successful male courtship and female receptivity. These findings demonstrate sexually dimorphic and state-dependent control of locomotor activity by pC1 neuronal activity and DH44 modulation.

## Introduction

A long-standing question in neuroscience is how sexually dimorphic behaviors are generated. In both invertebrates and vertebrates, a few quantitative differences of the nervous system, especially in the interneurons, are found in the 2 sexes of the same species establishing the neural basis of sexually dimorphic behaviors [1]. In *Drosophila*, the neuronal substrates underlying sexual dimorphism are specified by the male-specific product encoded by fruitless and the sex-specific products encoded by doublesex (Dsx^M^ in males and Dsx^F^ in females) [2–4]. A subset of interneurons defined by *dsx* in the posterior brain region termed pC1 in both sexes have been found to control dimorphic sexual and aggressive behaviors [5–9]. There are ~60 Dsx^M^-positive pC1 neurons in males, but only ~6 Dsx^F^-positive counterparts in females [10,11]. These pC1 neurons integrate multiple sex-related sensory inputs and encode internal states in both males and females [7,10,12]. The huge differences in the numbers and arborizations of pC1 neurons in males and females provide the circuit basis underlying sexually dimorphic behaviors. Here, we discover a neuromodulatory mechanism for sexual dimorphism, in which a neuropeptide functions in the established dimorphic pC1 neurons to specify dimorphic locomotor activities in males and females for efficient sexual behaviors.

## Results and Discussion

**Fig. 1.**
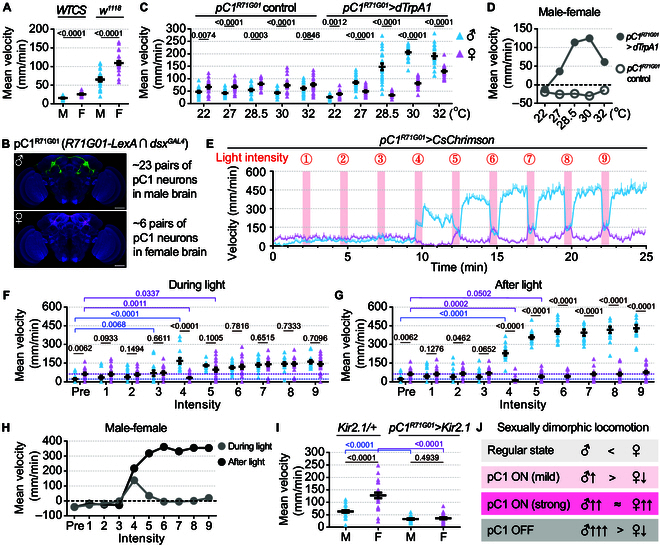
Sexually dimorphic locomotor activity in regular and aroused states. (A) Daily mean velocity in males and females of 2 wild-type strains. *n* = 17 for *WTCS* and *n* = 24 for *w^1118^*. (B) Image registration of pC1^R71G01^ neurons in male and female brains. Scale bars, 30 μm. (C) Daily mean velocity in males and females with thermogenetic activation of pC1^R71G01^ neurons at different temperatures. *n* = 20 to 24 for each. (D) Sex differences in daily mean velocity (male minus female) at each temperature. (E) The average velocity per 5 s for both sexes of *pC1^R71G01^>CsChrimson* flies during an intensity titration experiment. Each pink bar indicates a 30-s photostimulation. The first 2 min of the experiment represents the baseline phase. (F and G) The quantification of mean velocity per minute for both sexes of *pC1^R71G01^>CsChrimson* flies during (F) and after (G) photostimulation. *n* = 15 males and *n* = 21 females. (H) Sex differences in mean velocity (male minus female) at baseline and each light-intensity point. (I) Daily mean velocity in males and females with inactivation of pC1^R71G01^ neurons at 25 °C. *n* = 24 for each. (J) Summary of sexually dimorphic locomotion patterns in regular and aroused states. *P* values are indicated. For details of statistics, see the Supplementary Materials.

To compare locomotor activity in male and female flies, we firstly assayed spontaneous locomotion in 2 wild-type strains (*WTCS* and *w^1118^*) for 24 hours. We found that isolated males displayed significantly lower locomotor activity than isolated females (Fig. 1A). We next set out to compare locomotor differences in sexually aroused males and females by artificially activating sex-promoting pC1 neurons. We used an intersectional strategy as done previously (*dsx^GAL4^* and *R71G01-LexA*) [8,13] to label homologous pC1 neurons in both sexes of the same genotype. Such intersection labeled ~23 neurons per hemisphere in the male brain and ~6 neurons per hemisphere in the female brain, hereafter referred to as pC1^R71G01^ (Fig. 1B). We firstly expressed the *Drosophila* temperature-sensitive cation channel dTrpA1 in pC1^R71G01^ neurons for activation and set the temperatures as 5 gradients from 22 to 32 °C, where 22 °C represented the baseline. While control males consistently showed lower activity than control females at various temperatures, males with pC1^R71G01^ neurons being activated displayed significantly higher activity than females with activated pC1^R71G01^ neurons at each temperature (Fig. 1C and D and Fig. S1A and B).

Because of the lower precisions in the time resolution of thermogenetic protocols, we further drove the expression of red-shifted channel CsChrimson in pC1^R71G01^ neurons for optogenetic activation using a constant red light with 9 increasing intensities (level 1 to 9; Fig. 1E). We found that moderate activation (level 4) of pC1^R71G01^ neurons increased male locomotion and decreased female locomotion such that males are more active than females (Fig. 1F), which is generally consistent with the dTrpA1-mediated activation experiments. However, we also found distinct effects during and after optogenetic activation. While strong activation (>level 4) promoted both male and female locomotion to comparable levels during the “ON” phases (Fig. 1F and H), we observed a remarkably enhancement of male locomotion but a reduction of female locomotion following the light “OFF” (Fig. 1G and H), which may reflect distinct internal states in males and females. We propose that the long-term dTrpA1 activation may correspond to a summed effect of both “ON” and “OFF” phases from optogenetic activation.

To investigate whether the regular activity of pC1 neurons is involved in locomotor control, we silenced pC1 neurons by expressing the inwardly rectifying potassium channel Kir2.1 and observed similarly low levels of locomotion in both males and females (Fig. 1I and Fig. S1C and D). Together, these experiments demonstrate that pC1 neurons play crucial roles in regulating the sexually dimorphic locomotor activity in both sexually isolated and aroused males and females (Fig. 1J).

**Fig. 2.**
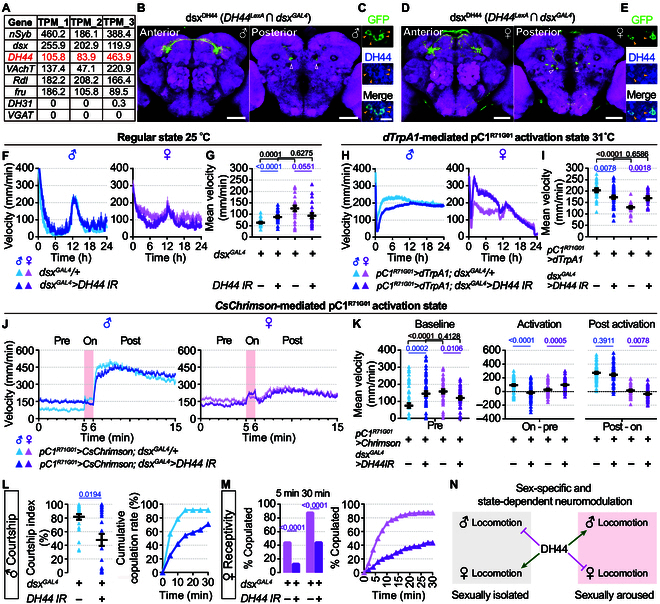
Neuropeptide DH44 functions in a sex-specific and state-dependent manner to regulate dimorphic locomotion and sexual behaviors. (A) Representative gene expression in male pC1 neurons from RNA sequencing. Three replicates were performed. (B to E) Confocal images showing anterior (left) and posterior (right) parts of dsx^DH44^ neurons in a male brain (B) and a female brain (D). Open arrowheads denote cell bodies of pC1 subsets. Scale bars, 50 μm. (C and E) Colocalization of dsx^DH44^ neurons and anti-DH44. Solid yellow arrowheads denote DH44-positive cells. Scale bars, 10 μm. (F and G) Twenty-four-hour locomotion profile (F) and mean velocity (G) with *DH44* knockdown in *dsx^GAL4^* neurons in males and females. *n* = 23 for each. (H and I) Twenty-four-hour locomotion profile (H) and mean velocity (I) for males and females during dTrpA1-mediated activation of pC1^R71G01^ neurons with *DH44* knockdown in *dsx^GAL4^* neurons. *n* = 24 and 41 males and *n* = 22 and 18 females from left to right. (J and K) Locomotor activity for males (J, left) and females (J, right) during CsChrimson-mediated photoactivation of pC1^R71G01^ neurons with *DH44* knockdown in *dsx^GAL4^* neurons, which were quantified in (K). The pink bar indicates a 1-min photostimulation. (K) Mean velocity in 5 min before photostimulation (left), and velocity changes during or after photostimulation (right). Two minutes after photostimulation was used for analysis. *n* = 58 males for each and *n* = 45 and 47 females from left to right. (L) Courtship index in 10 min (left) and cumulative copulation rate within 30 min (right) for males with *DH44* knockdown in *dsx^GAL4^* neurons at 25°C. *n* = 24 for each. (M) Copulation percentage within 5 and 30 min (left) and cumulative receptivity curve (right) for females with *DH44* knockdown in *dsx^GAL4^* neurons at 25°C. *n* = 96 for each. (N) Summary of the sex-specific and state-dependent modulation of locomotor activity by DH44. GFP, green fluorescent protein.

To identify molecular markers for pC1 neurons, we isolated male pC1 neurons for RNA sequencing and found that a neuropeptide-encoding gene *diuretic hormone 44* (*DH44*) was highly expressed in these cells (Fig. 2A). Intersection of the 2 knockin drivers, *dsx^GAL4^* and *DH44^LexA^*, labeled ~6 pairs of pC1 neurons in males and ~2 pairs in females (Fig. 2B and D). pC1 neurons were also labeled from intersection of 2 other knockin drivers, *dsx^LexA^* and *DH44^GAL4^* (Fig. S2A and B), as well as a split *GAL4* of *DH44^AD^* and *dsx^DBD^* (Fig. S2C and D). Double staining with anti-DH44 antibody confirmed that at least 4 of the 6 labeled male cells and the 2 female cells indeed colocalized with DH44 (Fig. 2C and E).

We next used RNA interference against *DH44* to investigate its potential role in regulating male and female locomotion. The effectiveness of the *UAS-DH44IR* line was verified by the lethal effect during the pupal stage under the control of *actin-GAL4* and the negative immunostaining signal with anti-DH44 using the *DH44^GAL4^* driver (Fig. S3). Interestingly, we observed a sex-specific effect of DH44. Knocking down *DH44* driven by *dsx^GAL4^* increased male locomotion but decreased female locomotion such that these males and females showed indistinguishable levels of locomotion (Fig. 2F and G). In contrast, knocking down *choline acetyltransferase* (*ChAT*) that encodes the biosynthetic enzyme for acetylcholine in *dsx^GAL4^* neurons reduced male and female locomotion to similarly low levels (Fig. S4A). Given that strong activation of pC1^R71G01^ neurons dramatically increased locomotion in both sexes, we wondered how DH44 was involved. To our surprise, after knocking down *DH44* in *dsx* neurons at a state of activating pC1^R71G01^ neurons via dTrpA1, male flies locomoted much more slowly, while female flies locomoted much faster (Fig. 2H and I). These data reveal that the already high locomotor activity resulting from intense activation of pC1^R71G01^ neurons is promoted by DH44 in males but suppressed by DH44 in females. These results indicate a state-dependent function of DH44, in addition to its sex-specific function.

To further confirm the above findings, we optogenetically activated pC1^R71G01^ neurons (intensity level 7) for 1 min in solitary males or females and continued to record locomotion for 10 min after light went off. As expected, *pC1^R71G01^>CsChrimson* males showed a robust increase in locomotor activity during photostimulation and went much higher after light offset. Furthermore, males with additional *dsx^GAL4^*-driven knockdown of *DH44* showed higher activity in baseline but no enhancement during photostimulation stage and a much less enhancement during the 2-min stage after photostimulation (Fig. 2J and K). In contrast, *pC1^R71G01^>CsChrimson* females moved slightly faster during photostimulation and returned to the baseline levels within a short time after light offset. However, *DH44*-knockdown females showed a lower locomotor activity in baseline, a higher activity during activation and decayed rapidly after it (Fig. 2J and K). Consequently, knockdown of *DH44* driven by *dsx^GAL4^* significantly reduced both male courtship (Fig. 2L) and female receptivity (Fig. 2M). Moreover, knocking down *DH44* specifically in pC1 neurons, but not vpoDN neurons crucial for vaginal plate opening, dramatically reduced female receptivity (Fig. S5).

Together, these results indicate that DH44 inhibits male locomotion but promotes female locomotion when they are sexually isolated, but, inversely, DH44 promotes male locomotion but inhibits female locomotion when they are sexually aroused with pC1 activation (Fig. 2N). The persistently heightened locomotor activity observed in males, but not females, following pC1 activation may indicate distinct internal states encoded by the sexually dimorphic pC1 neurons, which may be beneficial for both male and female reproductive behaviors. We speculate that DH44 functions in a subset of pC1 neurons, and perhaps in other neurons too, to play sex-specific and state-dependent modulatory roles via its 2 receptors. In most animal species, males command active locomotion to follow and court females with an elaborate ritual, while females are more passive in such a ritual and often reduce locomotion when deciding to accept a courting male. The male-specific and state-dependent modulation of locomotion by DH44 makes males more active and sensitive to potential mates, while saving energy without them. In contrast, the female-specific and state-dependent regulation by DH44 promotes locomotion if not being courted, probably for food and/or oviposition searching, and reduces locomotion for copulation if satisfied with male courtship.

It is worth noting that functional counterparts for both the DH44 peptide (corticotropin-releasing factor) and pC1 neurons (ventrolateral subdivision of the ventromedial hypothalamus) exist in mammals [14,15]. Thus, the sex-specific and state-dependent neuromodulation we discovered in flies could also apply to high-order animals. Future studies would reveal how DH44 exerts the sex-specific and state-dependent functions in pC1 neurons in molecular and cellular details to coordinate male and female innate behaviors.

## Materials and Methods

Flies were raised in a 22 or 25 °C environment with 60% humidity and a 12-h:12-h light:dark cycle. All the tested flies were group-housed virgins (∼20 single-sex flies per vial) between the ages of 5 to 7 d after eclosion without specifically mentioned. *Canton-S* and *w^1118^* flies were used as wild-type strains. Detailed methods, full genotypes of tested flies, data analysis were described in the Supplementary Materials.

## Data Availability

All data are provided in the figures and the Supplementary Materials. Resources and reagents are available upon reasonable request.

## Supplementary Materials

Supplementary 1 Supplementary Materials and Methods Supplementary References Figs. S1 to S5
