# Supplementary material for: Sex-Specific and State-Dependent Neuromodulation Regulates Male and Female Locomotion and Sexual Behaviors
Source: Research (Wash D C). 2024 Feb 22;7:0321. doi: 10.34133/research.0321 (PMC10882504; doi:10.34133/research.0321)
Supplement: Supplementary 1 — Supplementary Materials and Methods Supplementary References Figs. S1 to S5 [file research.0321.f1.pdf]

Supporting Information for

Sex-specific and state-dependent neuromodulation regulates male and female locomotion and sexual behaviors

Xinyu Jiang[1], Mengshi Sun[1], Jie Chen[1], Yufeng Pan[1,2*]

Affiliations

[1]The Key Laboratory of Developmental Genes and Human Disease, School of Life Science and Technology, Southeast University, Nanjing, 210096, China.

[2]Co-innovation Center of Neuroregeneration, Nantong University, Nantong, 226019, China.

[*]Correspondence to: pany@seu.edu.cn (Y.P.)

This PDF file includes:

Supplementary Materials and Methods

Supplementary References

Supplementary Figures S1-S5

Supplementary Materials and Methods

Fly strains

Flies were raised in a 22°C or 25°C environment with 60% humidity and a 12hr:12hr light: dark cycle. Each cross used 20 virgin females with half males and was flipped every three days. In this study, all the tested flies were group-housed virgins (~20 single-sex flies per vial) between the ages of 5-7 days after eclosion without specifically mentioned and transferred to fresh food the day before the experiments. Males and females were operated similarly. *Canton-S* and w^{1118} flies were used as wild-type strains shown in Figure 1A. *UAS-FRT-stop-FRT-CsChrimson* was from Dr. Fang Guo; $DH44^{GAL4}$ and $DH44^{LexA}$ were from Dr. Yi Rao [1]. $DH44^{AD}$ was generated in this study. *8xLexAop-FLP* (#55819), *UAS-FRT-stop-FRT-Kir2.1*^{eGFP} (#67686) and *R71G01-LexA* (#54733) were from Bloomington Drosophila Stock Center (BDSC). RNAi lines were from Tsinghua Fly Center (THFC) at the Tsinghua University. *UAS-FRT-stop-FRT-dTrpA1*, dsx^{GAL4} [2] and dsx^{DBD} [3]were used as previously. Flies with the genotype of w^{1118}*; UAS>>dTrpA1/+; 8xLexAop-FLP,* dsx^{GAL4}/+ and w^{1118}*; UAS>>dTrpA1/R71G01-LexA; 8xLexAop-FLP,* dsx^{GAL4}/+ were used for Figure 1C and 1D; w^{1118}*; UAS>>CsChrimson/R71G01-LexA; 8xLexAop-FLP,* dsx^{GAL4}/+ was used for Figure 1B, 1E-1H; w^{1118}*; UAS>>Kir2.1/+; 8xLexAop-FLP,* dsx^{GAL4}/+ and w^{1118}*; UAS>>Kir2.1/R71G01-LexA; 8xLexAop-FLP,* dsx^{GAL4}/+ were used for Figure 1I; w^{1118}*; UAS>>CsChrimson/*$DH44^{LexA}$*; 8xLexAop-FLP,* dsx^{GAL4}/+ were used for Figure 2B-2E; w^{1118}*; UAS>>dTrpA1/R71G01-LexA; 8xLexAop-FLP,* dsx^{GAL4}/+ and w^{1118}*; UAS>>dTrpA1/R71G01-LexA; 8xLexAop-FLP,* dsx^{GAL4}*/UAS-DH44 RNAi* were used for Figure 2H and 2I; w^{1118}*; UAS>>CsChrimson /R71G01-LexA; 8xLexAop-FLP,* dsx^{GAL4}/+ and w^{1118}*; UAS>>CsChrimson/R71G01-LexA; 8xLexAop-FLP,* dsx^{GAL4}*/UAS-DH44 RNAi* were used for Figure 2J and 2K; and w^{1118}*;; 8xLexAop-FLP,* dsx^{GAL4}/+ and w^{1118}*;; 8xLexAop-FLP,* dsx^{GAL4}*/UAS-DH44 RNAi* were used for Figure 2F, 2G, 2L and 2M.

Generation of $DH44^{AD}$

The $DH44^{AD}$ knock-in line was generated using similar strategy as previously described [1]. In summary, the coding sequence of *p65AD*, which was amplified from an Addgene plasmid #26234, along with the DNA sequence that was removed from $DH44^{attP}$ flies, were inserted into the attB-GMR-miniwhite vector. The knock-in construct was confirmed through sequencing and was integrated into the attP site of $DH44^{attP}$ flies, after which the selection markers were removed using the Cre/loxP system.

Generation of anti-DH44 antibody

The rabbit polyclonal antibodies against the *Drosophila* DH44 protein were generated by Wuhan Abclonal Biotechnology. At least two specific pathogen-free Japanese White Rabbits were immunized with chemosynthetic polypeptides (DDGDNEGEDSYNDVGTEGVG, 281–300 amino acids of DH44). After a successful antiserum detection by ELISA, the rabbits were sacrificed, and their serum was affinity purified using the same antigen to obtain the final antibodies.

Immunostaining

The brains of 7-day-old flies were dissected in Schneider's insect medium (Thermo Fisher Scientific, 21720). They were then fixed in 4% paraformaldehyde in phosphate-buffered saline (PBS) for 1h on ice. After being washed 4 times for 15 minutes each in PAT3, which consisted of 0.5% (v/v) Triton X-100 and 0.5% (w/v) bovine serum albumin in PBS, the brains were blocked in 3% normal goat serum (NGS) for 1h at room temperature. Brains were subsequently incubated with the primary antibody solution which had been diluted in 3% NGS overnight at 4°C, following a 4-times 15-min wash in PAT3 at room temperature. The primary antibodies used here were rabbit anti-GFP paired with nc82, which were diluted at a ratio of 1:1000 and 1:100 or rabbit anti-DH44 paired with mouse anti-GFP, which were diluted at a ratio of 1:500 and 1:1000. Finally, the brains were incubated with the secondary antibody solution using

the same preparation way for 1-2 days at 4°C. All secondary antibodies used here (donkey anti-rabbit or mouse IgG conjugated to Alexa 488 and donkey anti-rabbit or mouse IgG conjugated to Alexa 555) were diluted at a ratio of 1:500. After being washed 4 times thoroughly in PAT3 at room temperature, the brains were mounted by the VECTASHIELD mounting medium for imaging. Whole brains were imaged at 10× and cell bodies were imaged at 20× lens (Olympus) using confocal microscope (Zeiss, LSM900) with a resolution of 1024 x 1024 pixels. All imaging data were analyzed using Fiji.

Locomotion assay

Two-layer chambers with a diameter of 2cm (24-well) were used to test spontaneous locomotion for both sexes. Individual flies were gently aspirated into the upper layer of the chambers with light color food filled into the lower layer. In all experiments, loaded flies were placed directly into an incubator pre-set with the specific temperature (22°C, 27°C, 28.5°C, 30°C or 32°C for dTrpA1-mediated activation experiments; 25°C for others) at ZT0 of the test day, and a 24-hour movie was captured with full exposure to visible light. A thin film was removed upon videotaping in all experiments to make the initial locomotion more comparable. The walking of flies was tracked by the ZebraLab software system (ViewPoint Life Sciences) with data output at 1min resolution. The average speed per 5 minutes was calculated to show the locomotor activity profile within 24 hours and the mean velocity of each fly over a 24-hour period was also calculated to facilitate comparison.

Male courtship and female receptivity assay

Two-layer chambers with a diameter of 1cm (48-well) were used to test both male courtship and female receptivity. These two behaviors have similar ways of detection, with a difference that tested males were paired with 4 to 6-day-old wild-type virgin females in the male courtship assay while tested females were paired with 4 to 6-day-

old wild-type virgin males in the female receptivity assay. On the test day, a tester and its single target were loaded individually into the chambers with gentle aspiration through a hole in the top plate. They were separated by a plastic transparent thin film in the middle of two acrylic plates until the videotaping began and last for 30 min at 25°C.

Male courtship was evaluated by two parameters including 'courtship index' and 'cumulative copulation rate'. The courtship index was calculated as the fraction of time the flies spent performing any step of courtship within 10 min after courting initiation. The cumulative copulation rate was calculated as the cumulative proportion of males who copulated successfully per 5 minutes throughout the 30-min movies. For female receptivity, the cumulative percentage of copulation was counted per 2 minutes throughout the 30-min movies, and the percentage at 5min and 30min were redrawn as a histogram for further comparison. Both males and females were scored manually with the usage of a LifeSongX software.

Optogenetic assay

For optogenetic experiments, crosses were reared under standard conditions at 25°C but newly eclosed flies were transferred to retinal food which was made by adding all-trans-retinal to standard fly food, with a final concentration of 1 mM and kept in the dark until the test. A LED panel with 4 channel (Sea-light, GB200X300-RGBIR) was fixed on the stage to provide photostimulation with different wavelengths of light at various intensities which was adjusted by an externally dimmable LED driver (Sea-light, GP-245-4C). The light switch was controlled by a custom-designed PLC (Uni Electronic Tech, UN2070-0606RT). Two-layer behavioral chambers with a diameter of 2cm (24-well) were placed on the surface of the LED panel. Each chamber held a single fly, which was gently aspirated into the upper layer and transparent food (5% sucrose and 2% agarose) in the lower layer to reduce heat caused by photostimulation. Flies were recorded under an infrared backlight by a camera (HIK VISION) equipped

with an infrared filter at 12 fps to allow the visualization of the flies in the dark and avoid detection of light from the photostimulation.

All optogenetic experiments in our study used constant illumination with the wavelength at 620nm. Flies were loaded and acclimated in the setup for 1 hour before the test. In the experiment shown in Figure 1, we tested the same set of flies with 9 increasing intensities of 30s photostimulation with a 2-min inter-stimulus interval after a 2-min pre-stimulation baseline. The 9 levels of photostimulation are named from 1 to 9, whose intensities correspond to 0.48, 0.59, 0.70, 0.81, 0.92, 1.04, 1.15, 1.26, 1.37 $\mu W/mm^2$, measured by an optical power meter (Fastlaser Tech) placed on the board with transparent food. For the experiment shown in Figure 2, 1-min photostimulation at level 7 (1.15 $\mu W/mm^2$) was given after a 5-min pre-stimulation recording followed by a 10-min post-stimulation recovery. These experiments were conducted within ZT2-ZT8. The mean velocity of each stage (pre-, during- and post-stimulation) were calculated.

Cell picking, RNA sequencing and analysis

We initially used a *P1-splitGAL4* (*R15A01-AD; R71G01-DBD*) driving *UAS- tdTomato* for manual cell picking of P1/pC1 neurons. In brief, $tdTomato^+$ cells were manually picked from dissected fly brains under a fluorescence microscope following a previous protocol [4], with a minimum of 50 $tdTomato^+$ cells for each sample. The picked cells underwent three washes before being loaded into a lysis buffer to ensure sample purity. RNA was extracted by the PicoPure RNA Isolation Kit (KIT0204, Thermo Scientific) and converted to cDNA by the Ovation RNA-Seq System V2 (NuGEN, #7102-A01). The Initial quality check was performed using qPCR with the *dsx* gene as a positive control and the *repo* gene as a negative control, before proceeding to DNA fragmentation and the final adaptor ligation for sequencing. The cDNA libraries were sequenced by Illumina Hiseq 2500 platform. The sequenced raw data were first pre-processed to remove low-quality reads, adaptor sequences and amplification primers.

Reads were mapped to *Drosophila* genome and the mapped reads were selected for further analysis. Gene expression was quantified using TPM (transcripts per million). This preliminary data identified *DH44* as a potential molecular marker for a subset of pC1 neurons for further studies (Figure 2A).

Statistical analyses

Statistical analyses were performed by GraphPad Prism 9. For comparison between two groups, Unpaired t-tests (Figure 1A, 1C and 1I) or Mann-Whitney U-tests (1F, 1G, 2G, 2I, 2K and 2L) were conducted depending on whether the data was normally distributed. To compare the effects of different stimuli on the locomotion of the same individual in Figure 1F and 1G, Paired t tests were applied to females in Figure 1F, while Wilcoxon matched-pairs signed rank tests were applied to others on account of the non-normal distribution. To compare female copulation percentage in Figure 2M, Chi-square test was used. P values are indicated in each figure, and $P > 0.05$ was considered to be not significant. The sample sizes (n number) for each experiment are indicated in the figure legends.

Supplementary References

1. Deng, B., Li, Q., Liu, X., Cao, Y., Li, B., Qian, Y., Xu, R., Mao, R., Zhou, E., Zhang, W., et al. (2019). Chemoconnectomics: Mapping Chemical Transmission in Drosophila. Neuron *101*, 876-893 e874.
2. Pan, Y., Meissner, G.W., and Baker, B.S. (2012). Joint control of Drosophila male courtship behavior by motion cues and activation of male-specific P1 neurons. Proc Natl Acad Sci U S A *109*, 10065-10070.
3. Han, C., Peng, Q., Sun, M., Jiang, X., Su, X., Chen, J., Ma, M., Zhu, H., Ji, X., and Pan, Y. (2022). The doublesex gene regulates dimorphic sexual and aggressive behaviors in Drosophila. Proc Natl Acad Sci U S A *119*, e2201513119.
4. Li, C., Li, X., Bi, Z.H., Sugino, K., Wang, G.Q., Zhu, T., and Liu, Z.Y. (2020). Comprehensive transcriptome analysis of cochlear spiral ganglion neurons at multiple ages. Elife *9*.

Supplementary Figures and Legends

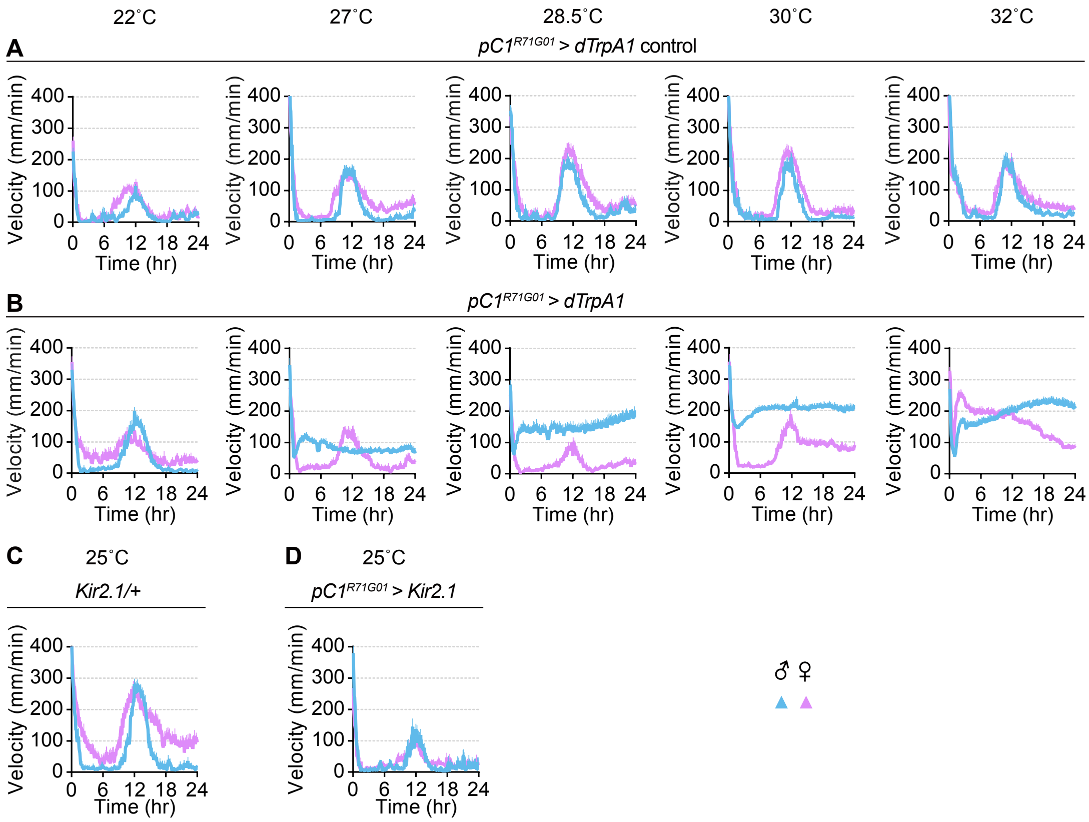

Figure S1. pC1-mediated locomotion patterns are sexually dimorphic.

(A and B) 24-hour locomotion profiles of males and females during thermogenetic activation of $pC1^{R71G01}$ neurons at different temperatures, which were quantified as daily mean velocity in Figure 1C. n=20-24 for each. (C and D) 24-hour locomotion profiles of males and females with $pC1^{R71G01}$ neurons being silenced via Kir2.1 at 25°C, which were quantified in Figure 1I.

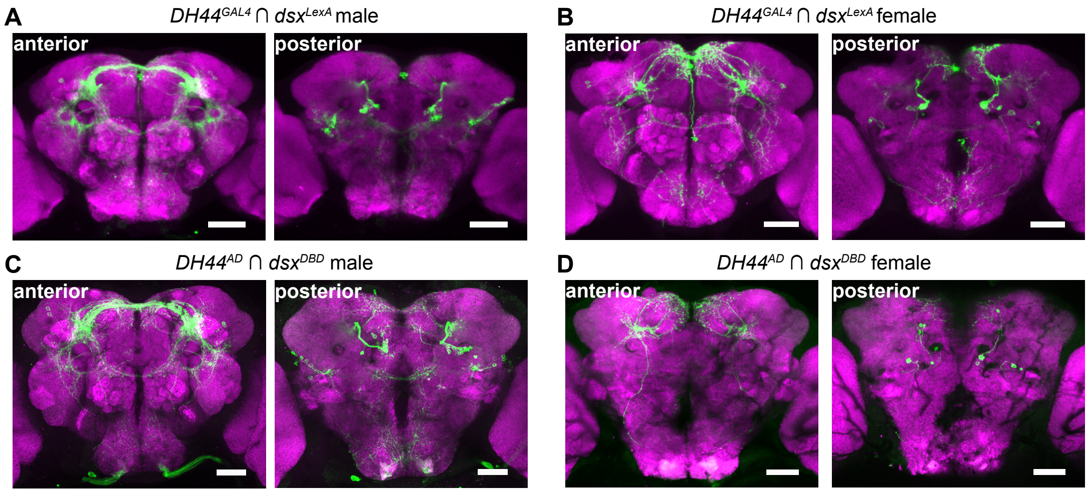

Figure S2. Overlapped expression of *DH44* and *dsx* neurons.

(A and B) Confocal images showing brain anterior (left) and posterior (right) parts of pC1^{DH44} neurons from a male (A), and a female (B) with the genotype of *dsx^{LexA}*, *DH44^{GAL4}*, *UAS-FRT-stop-FRT-CsChrimson*, and *LexAop-FLP,* co-stained with anti-GFP (green) and anti-nc82 (magenta). (C and D) Confocal images showing anterior (left) and posterior (right) parts of *UAS-myrGFP/+; DH44^{AD}/dsx^{DBD}* male (C) and female (D) brains, co-stained with anti-GFP (green) and anti-nc82 (magenta). Scale bars: 50 µm.

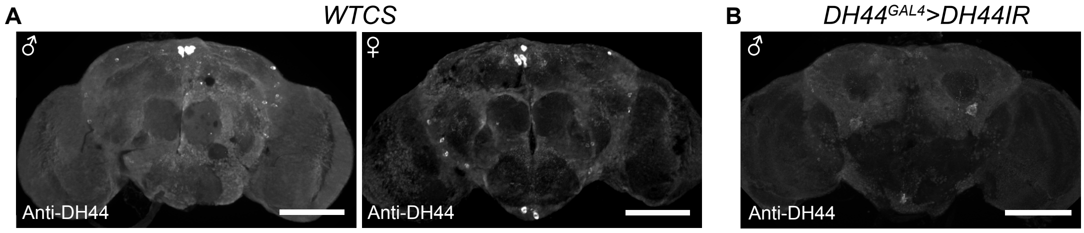

Figure S3. Validation of *DH44* RNAi efficiency.

(A) Confocal images showing DH44 immunostaining from wild-type male (left) and female (right) brains. DH44 expression is most strong in the Pars Intercerebralis (PI) region. Scale bars: 100 µm. (B) Confocal image showing DH44 immunostaining from a male fly with RNAi-mediated *DH44* knockdown under the *DH44^{GAL4}* driver. Scale bar: 100 µm.

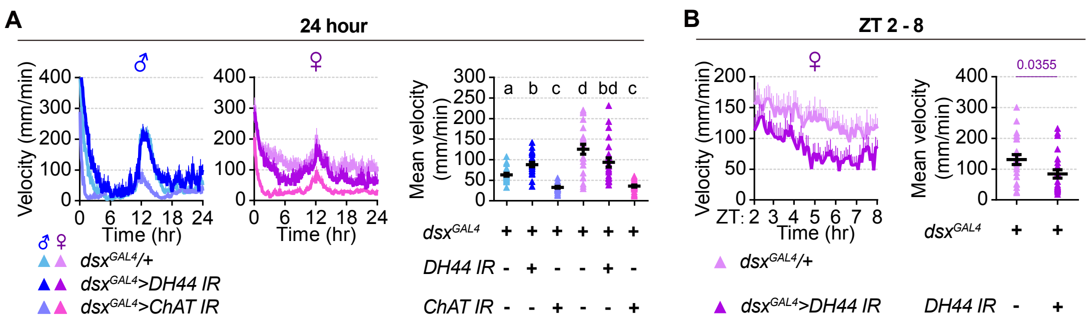

Figure S4. Knocking down *ChAT* in *dsx* neurons reduces both male and female locomotion.

(A) 24-hour locomotion profile (left) and mean velocity (right) with *DH44* and *ChAT* knockdown in dsx^{GAL4} neurons in males and females, corresponding to data in Figure 2F and 2G. n=23 for each. All variables that have different letters are significantly different ($p < 0.05$), and all variables with the same letter are not significantly different, Mann-Whitney U-tests. (B) Detailed locomotion profile from ZT2 to ZT8 (left) and mean velocity (right) with *DH44* knockdown in dsx^{GAL4} neurons in females, corresponding to female data in (A). Unpaired t-test.

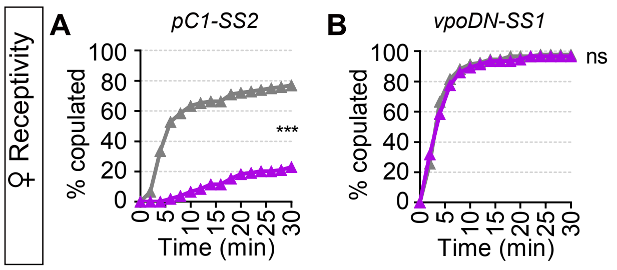

Figure S5. DH44 in pC1 neurons promotes female receptivity.

(A and B) 30-min receptivity profile of virgin females with *DH44* knockdown in pC1-SS2 neurons (A), and vpoDN-SS1 neurons (B) at 25°C. n=93-104. ***$p < 0.001$, Chi-square test.
